# Study of the protective effects of cosmetic ingredients on the skin barrier, based on the expression of barrier-related genes and cytokines

**DOI:** 10.1007/s11033-021-06918-5

**Published:** 2021-11-19

**Authors:** Wenyu Ding, Linna Fan, Yan Tian, Congfen He

**Affiliations:** 1 Cosmetics Department, College of Chemistry and Materials Engineering, Beijing Technology and Business University, No. 11 Fucheng Road, Haidian District, Beijing, 100048 China; 2 Air Force Medical Center, PLA, Beijing, 100142 China

**Keywords:** HaCaT, Sensitive skin, Cosmetic ingredients, Safety testing, Skin barrier

## Abstract

**Background:**

Sensitive skin is the result of a complex process that is closely linked to the damage of the skin barrier. There are no recognized methods for evaluating the efficacy of anti-allergy products.

**Methods:**

In this study, a model of skin barrier damage was created by treating HaCaT cells with 60 μg/ml of sodium dodecyl sulfate for 48 h. The protective effects of nine cosmetic ingredients, including oat extract (S1), on the skin barrier were investigated based on the gene expression levels of aquaporin3 (AQP3), filaggrin (FLG), caspase-14 (CASP14), and human tissue kallikrein7 (KLK7), as well as those of various interleukins (IL) and vascular endothelial growth factor (VEGF).

**Results:**

Among the nine ingredients, S1 had a good protective effect on the function of the skin barrier. It promoted the expression of AQP3, FLG, and CASP14, while inhibiting the expression of KLK7 in HaCaT cells, at a concentration of 0.06%. It also maintained IL-6, IL-8, and VEGF at appropriate levels while promoting the proliferation and differentiation of HaCaT cells.

**Conclusions:**

The above indicators allow for the preliminary establishment of a method to evaluate the efficacy of the barrier protection ability of sensitive skin.

**Supplementary Information:**

The online version contains supplementary material available at 10.1007/s11033-021-06918-5.

## Introduction

**Fig. 1:**
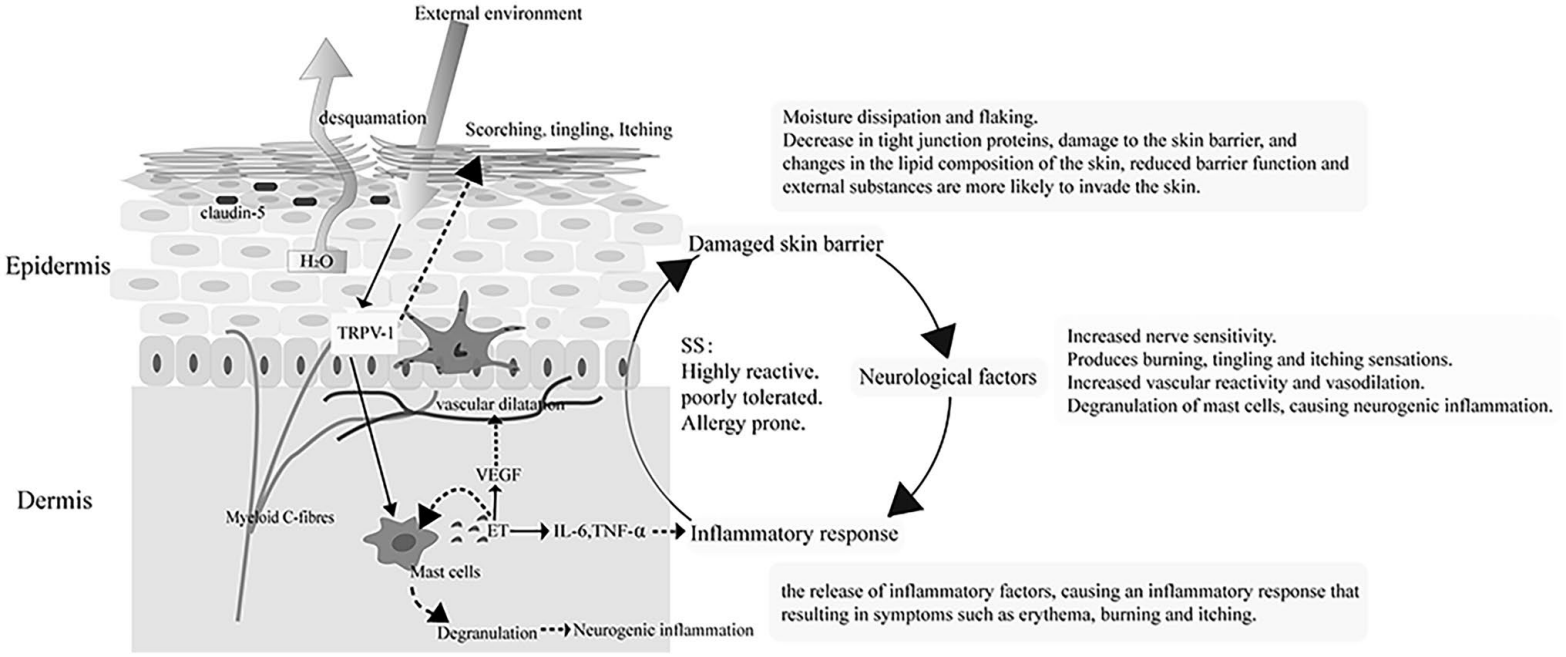
Causes of skin sensitivity

Sensitive skin (SS) refers to a state of hyper-reactivity of the skin under physiological or pathological conditions. It can be characterized by subjective symptoms such as burning, tingling, itching, and tightness, with or without objective signs such as erythema, capillary dilation, and desquamation [1]. The development of SS is a complex process involving barrier function, neurological factors, and inflammatory response [2–5], as shown in Fig. 1, wherein it is shown to be associated with high reactivity, poor tolerance, and susceptibility to allergy. Impaired skin barrier function is an important cause of skin sensitivity [6].

Aquaporin3 (AQP3) is involved in the uptake and secretion of substances from cells, contributing to the maintenance of elasticity and the repair of damage. Filaggrin (FLG) connects keratin fibers to maintain the flattened shape of keratinocytes and to form the keratin envelope. Caspase-14 (CASP14) is involved in the terminal differentiation of keratinocytes and assists FLG in forming the complete keratinocyte layer [7–10]. Human tissue kallikrein 7 (KLK 7) hydrolyses the bridging protein [11], which acts as a link between keratinocytes, resulting in impaired barrier function. The above four proteins are important for maintaining skin barrier homeostasis, with high expression of KLK 7 triggering severe skin flaking and low expression of the other three proteins, causing incomplete stratum corneum structure and skin barrier damage. The genes of these four proteins are thus known as skin barrier-related genes, and their expression can determine the health of the skin barrier, as well as indirectly indicate the protective or reparative capacity of a substance tested for its effects on the skin barrier.

The release of interleukin (IL), which is more sensitive to external stimuli, can lead to further damage of the skin barrier. Over-expression of vascular endothelial growth factor (VEGF) induces vasodilation, enhances endothelial permeability, damages blood vessels, and contributes to the development and persistence of inflammation. For these reasons, IL and VEGF are known to be skin barrier-related factors. Measuring the expression of cytokines such as IL can further indicate the extent of damage to the skin barrier, while inhibiting the expression of associated inflammatory factors can mitigate the SS caused by the inflammatory response, as well as reduce its damage to the skin barrier.

In this study, a model of skin barrier damage was created by using SDS to damage HaCaT cells, which were then used to investigate the protective effect of S1 and nine other cosmetic ingredients. Levels of AQP3 and those of other indicators were evaluated as key parameters for screening the efficacy of the cosmetic ingredients capable of maintaining the skin barrier. The study aimed to establish a primary experimental system for evaluating the effectiveness and barrier protection ability of sensitive skin.

## Material and methods

### Materials

In this study, nine ingredients that had demonstrated good in vitro biochemical activity in our laboratory, were selected, they have a good ability to scavenge free radicals and inhibit hyaluronidase. They were labeled: oat (*Avena sativa*) extract (S1), four stamen stephania (*Stephania tetrandra*) root (S2), stachyose (S3), erythritol (S4), ceramide 3 liposomes (S5), olive (*Olea europaea*) leaf extract (S6), compound anti-allergic itch-relief remedy (S7/8), and brown algae (Phaeophyceae) extract (S9).

HaCaT (immortalized human epidermal keratinocyte) cells were purchased from the Peking Union Medical College Centre (Beijing, China). DNA markers and real-time PCR components were purchased from Invitrogen Biotechnology (Thermo Fisher Scientific, Waltham, MA, USA). GelRed was obtained from Bio-Rad (Hercules, CA, USA). BD CAB Flex Set was purchased from BD Biosciences (Franklin Lakes, NJ, USA). TRizol was purchased from Ambion (Life Technologies, Carlsbad, CA, USA).

The main instruments employed included: an enzyme labeler (TECAN, Männedorf, Switzerland), a gel imaging analysis system (Alpha Innotech, San Leandro, CA, USA), an Accuri C6 flow cytometer (BD Biosciences), a PCR thermocycler (Eppendorf, Hamburg, Germany), a real-time fluorescence quantitative PCR system (Roche, Basel, Switzerland), and an electrophoresis instrument (Tanon Science & Technology Co., Ltd., Shanghai, China).

### Construction of a HaCaT cell model by SDS

HaCaT cells were cultured with reference to the method described by Cui et al. [12], and cell concentrations with OD values in the range of 1.0 ~ 1.4 were screened by the MTT assay for subsequent experiments. The cells were treated with different concentrations of SDS to determine the appropriate concentration of SDS (cell viability of 60–80%) to construct a skin barrier damage model.

### Effect of MTT assay samples on the activity and protection of HaCaT cells

Cells were incubated for 48 h with the sample solutions (experimental group), SDS solution (positive control group), or PBS (blank control group), after which their viability was determined using the MTT assay (Fortuneibo-tech Co., Ltd, Shanghai, China). Six sample concentrations with cell viability above 90% were selected for subsequent testing. Cells were incubated for 24 h with the sample solution after which SDS solution was added for another 24 h of incubation. OD values were measured to detect the protective effect of the samples on the HaCaT cell model. Three sample concentrations (Table 1) with cytoprotective effects were selected for subsequent experiments.

**Table 1:** The nine samples represented are ingredients, and their specific concentrations, which with cytoprotective effects

Liquid samples	Low concentration(v/v,%)	Medium concentration(v/v,%)	High concentration(v/v,%)
S1	0.02	0.04	0.06
S2	0.004	0.005	0.006
S7	0.2	0.25	0.3
S8	0.004	0.006	0.008

Solid samples	Low concentration(μg/ml)	Medium Concentration(μg/ml)	High concentration(μg/ml)
S3	200	250	400
S4	300	400	500
S5	200	250	300
S6	100	125	150
S9	0.3	0.4	0.5

The liquid samples are as follows: S1, oat extract; S2, four stamen stephania extract; S7 and S8, compound anti-allergic itch-relief remedy. The solid samples are as follows: S3, stachyose; S4, erythritol; S5, ceramide 3 liposomes; S6, olive leaf extract; S9, brown algae extract

### AQP3 and other barrier-related gene testing

Real-time fluorescence quantitative PCR was used to evaluate *AQP3*, *FLG*, *CASP14, and KLK7* gene expression. The experimental systems were all based on the method of CUI et al. [12], and the primer sequences are shown in Table 2.

**Table 2:** Primer sequences for real-time fluorescent quantitative PCR

Gene name		Primer sequences
AQP3		Upstream: 5′-AGATGCTCCACATCCGCTAC-3′
		Downstream: 5′-GGTTGATGGTGAGGAAACCA-3′
FLG		Upstream: 5′-TGACAGTCAGGGACACTCAGA-3′
		Downstream: 5′-GGTGTCTGGAGCCATCTCTT-3′
CASP14		Upstream: 5′-CCCAAGGTGTACATCATACAGG-3′
		Downstream: 5′-TCTTTGATGACCATCACAATCTC-3′
KLK7		Upstream: 5′-CCTGCTCAGTGGCAATCA-3′
		Downstream: 5′-CAGGTGACAGGTGTACTCAT-3′
GAPDH	House-keeping genes	Upstream: 5′-ACATAGGCGCTCACTGTTCTC-3′
		Downstream: 5′-GCCCAATACGACCAAATCC-3′
β-actin		Upstream: 5′-CCAACCGCGAGAAGATGA-3′
		Downstream: 5′-CCAGAGGCGTACAGGGATAG-3′

### IL and other cytokine assays

The effect of the treatment samples on the secretion of IL-6, IL-8, and VEGF by HaCaT cells was measured via cytokine flow assay. According to the instructions of the Human Soluble Protein Master Buffer Kit (BD Biosciences). After the samples were collected (in FCS 2.0 format), a standard curve was plotted and the data analyzed using the Cytometric Beads Array (CBA) dedicated analysis software, FCAP Array v1.0 (BD Biosciences).

### Statistical analysis

Statistical analysis was performed using IBM SPSS Statistics 22. Each sample was done in 3 parallel. Between-group test using independent samples t-test with one-way ANOVA, p < 0.05 indicates a statistically significant difference.

## Results

### Construction of a HaCaT cell model of SDS injury

The MTT method was used to detect the effect of SDS on the activity of HaCaT cells, and the results showed that SDS was not cytotoxic at concentrations below 30 μg/ml, while the cell viability dropped to a minimum above a concentration of 80 μg/ml. SDS (60 μg/ml) was chosen as the stimulant concentration in this experiment, and its cell viability was 60%.

### Effect of MTT assay samples on the activity and protection of HaCaT cells

The nine treatment samples were divided into two categories: liquid samples (S1, S2, S7, and S8) and solid samples (S3, S4, S5, S6, and S9), and the cytotoxicity of each sample was compared after incubation. The results showed that the nine samples were not cytotoxic, some potentially enhancing cell proliferation at low concentrations. Cell viability gradually decreased as the concentration of the samples increased until a point beyond which viability plateaued.

### Effect of samples on AQP3 gene expression in HaCaT cells

SDS (60 μg/ml) significantly reduced the expression of *AQP3* in HaCaT cells; while samples S1, S5, S6, and S8 promoted *AQP3* expression in HaCaT cells in a dose-dependent manner (Fig. S1). Within a certain range, the concentrations of these treatment samples were directly proportional to *AQP3* gene expression.

### Effect of samples on FLG and CASP14 gene expression in HaCaT cells

SDS had no significant effect on the expression of *FLG* gene in HaCaT cells, but significantly inhibited the expression of the *CASP14* gene (Fig. S2). Samples S1, S2, S3, S4, and S5 promoted the expression of *FLG* and *CASP14* genes, at higher concentrations.

### Effect of samples on KLK7 gene expression in HaCaT cells

SDS significantly inhibited the expression of *KLK7* gene in HaCaT cells (Fig. S3). Samples S1, S4, S6, S8, and S9 inhibited the expression of *KLK7* in HaCaT cells at high concentrations but promoted it at low concentrations.

### Effect of samples on IL-6 secretion by HaCaT cells

The results of the experiments are shown in Fig. S4. All nine treatment samples promoted IL-6 secretion by HaCaT cells, but at concentrations below 20 pg/ml. This indicated that IL-6 could not be stimulated below minimal concentrations, despite the moderate enhancement of the proliferation and differentiation of these cells.

### Effect of samples on IL-8 and VEGF secretion by HaCaT cells

Samples S1, S2, S3, S4, and S5 showed dose-dependent promotion of IL-8 and VEGF secretion by HaCaT cells, and all nine samples showed consistent effects on IL-8 and VEGF expression (Fig. S5). Small amounts of IL-8 and VEGF promoted the proliferation of HaCaT cells.

## Discussion

A skin barrier damage model was created in HaCaT cells by incubating with 60 μg/ml of SDS and the cell survival rate was 60%. This induced changes in skin barrier-related genes and cytokine levels.

Aquaporins (AQP, aquaporin), located in cell membranes, are responsible for the transport of water and small molecules such as glycerol and urea. AQP3 is a member of the family of aquaporins predominant in the skin and play an important role in maintaining skin hydration, as well as regulating the proliferation, migration, and early differentiation of keratinocytes [13, 14]. Studies have shown that AQP3 knockout mice have impaired skin hydration, maintenance of elasticity, and repair of barrier function, and that glycerol administration alleviates this condition [15]. When cells are exposed to inflammatory or high osmolarity conditions, cellular AQP3 expression is upregulated in response to stress, as is the case in aged or atopic dermatitis skin [16–18]. In this study, samples S1, S5, S6, and S8 were found to promote the expression of *AQP3* in a dose-dependent manner; thus, emerging as potentially beneficial for the maintenance of the skin barrier.

The degradation of FLG in the epidermis is a natural process that maintains the moisture content and barrier structure of the stratum corneum. FLG also initiates the degradation of keratinocyte nuclei resulting in non-nucleated keratinocytes, hence is important for the terminal differentiation of keratinocytes, and thus the differentiation and formation of the epidermis [19]. CASP14 is a member of the cysteine aspartate-specific protease (caspase protease) family that is specific to the skin, and its activation is closely linked to keratinization of keratinocytes [20]. CASP14 is also a key enzyme for FLG production as it catalyzes the dephosphorylation of pro-FLG in the granular layer of the skin, to form FLG [21]. These processes are essential for regulating the composition of the skin surface barrier [22, 23] and are often used together as an indicator of the functional status of the skin barrier. During instances of external infection or irritation, CASP14 expression is affected and downregulated, leading to a decrease in substances that maintain the skin barrier structure, such as FLG and natural moisturizing factors. This results in impaired barrier function and adverse skin reactions such as dryness and flaking [24]. The effect of the samples used in this study, on the expression of *FLG* and *CASP14* genes in HaCaT cells was consistent. At higher concentrations, samples S1, S2, S3, S4, and S5 promoted the expression of both *FLG* and *CASP14,* while the others had no significant effect on the gene expression of the two proteins. SDS had no significant effect on the secretion of *FLG* by HaCaT cells, but significantly inhibited the expression of *CASP14*, indicating that SDS did not damage HaCaT cells by directly inhibiting the expression of *FLG* gene, but via inhibiting the expression of *CASP14* and thus the formation of FLG.

KLK7, a member of the human tissue kallikreins (KLKs) family, is produced by keratinocytes in the granular layer and secreted into the intercellular spaces of the stratum corneum [25]. KLK7 hydrolyses corneodesmosin and desmocollin, the main components of bridging proteins that connect keratinocytes to each other [11], causing degradation of the bridging proteins and loss of intercellular adhesion, resulting in flaking of the skin. This role of KLK7, in balance with the rate of differentiation of keratinocytes in the granular layer, maintains the vitality and homeostasis of the stratum corneum. However, when KLK7 activity is too high, severe flaking of the skin and impaired barrier function can be triggered. Studies have shown that KLK7 levels are significantly increased in the stratum corneum of irritated [26] or inflamed [27] skin and that topical application of targeted active agents such as salicylic acid [10] can downregulate KLK7 expression and thus alleviate skin barrier damage. In this study, S1, S4, S6, and S9 inhibited KLK7 expression at high concentrations, while the study samples at low concentrations promoted KLK7 expression, indicating that the effective concentration of the active substance should be appropriately increased if the purpose of the cosmetic agent is to maintain the skin barrier.

Damage to the skin barrier and corresponding changes in the gene expression of its associated proteins can stimulate an inflammatory response, causing the release of inflammatory factors. IL-6 is a functionally complex cytokine involved in cell proliferation, differentiation, inflammation, and immune regulation. During pathological conditions, IL-6 overexpression occurs, causing a local inflammatory response and leads to tissue damage [28, 29]. However, during normal physiological conditions, IL-6 is maintained at low levels to stimulate keratinocyte growth, and moderately promote their proliferation and differentiation [30], thereby helping to maintain normal skin metabolism [31]. This study found that addition of nine treatment samples promoted a limited amount of IL-6 secretion in HaCaT cells, but the IL-6 concentration was below 20 pg/ml, suggesting that the level was low enough not to cause an inflammatory response in an organism.

IL-8 is a chemotactic cytokine that promotes the chemotaxis of lymphocytes and neutrophils, leading to an inflammatory response through a series of actions and is hence considered an important facilitator of inflammation [32]. The small amount of IL-8 expressed in keratinocytes also has a pro-proliferative and auto-chemotactic effect. According to the studies on the expression of IL-8 in the healing process of human skin tissues after pricking and incision [33] and during skin transplantation in rabbits [34], maintaining a certain amount of IL-8 is important for the healing process after skin injury. Wang et al. [31] observed that when total peony glycosides acted in a concentration range that inhibited IL-8 expression in cell supernatants, they also inhibited cell proliferation, thus indirectly confirming the promotion of keratinocyte proliferation and chemotaxis by the expression of a small amount of IL-8.

VEGF is the most effective factor in promoting the growth of vascular endothelial cells, acting on them by binding to corresponding receptors to promote their proliferation and angiogenesis. The small amount of VEGF expressed in normal skin keratinocytes helps to maintain the normal density and permeability of blood vessels, facilitating nutrient transport and skin metabolism [35]. Overexpression of VEGF has been shown to lead to local vascular hyperplasia and hyper-permeability, which can be used as a biochemical indicator to evaluate the efficacy of drugs in psoriasis [36] and other skin lesions. Contrarily, reducing VEGF levels can effectively diminish its binding to vascular receptors and thus reduce microvascular hyperplasia [37]. In addition, superficial blood vessels are susceptible to external stimuli, which may induce vascular hyper reactivity and release of inflammatory mediators, sequelae closely associated with SS [38]. In this study, S5 demonstrated marked promotion of VEGF, which could potentially cause an increase in vascular reactivity and thus SS, implying cosmetics containing this ingredient may be unsuitable for people with SS. Several other ingredients showed weaker stimulation of VEGF, while S6 and S9 inhibited its expression.

## Conclusion

A model for damaged skin barrier was created by using HaCaT cells exposed to 60 μg/ml SDS. This was used to detect changes in the gene expression of barrier-related proteins—*AQP3, FLG, CASP14, KLK7*—and cytokines—IL-6, IL-8, and VEGF— which together could be used as indicators for evaluating the efficacy of cosmetic agents in protecting the skin barrier. An ingredient capable of stimulating the expression of *AQP3, FLG,* and *CASP14*, while inhibiting the expression of *KLK7*, plus maintaining the levels of IL-6, IL-8, and VEGF in the appropriate concentration range, can be important in the protection of the skin barrier. It could be used as a cosmetic ingredient to achieve a soothing effect while maintaining the skin barrier.

## Supplementary Information

Below is the link to the electronic supplementary material.

Supplementary file1 (TIF 1662 KB) Effect of nine samples of different concentrations on AQP3 gene expression in HaCaT cells. The nine samples included both liquid and solid samples, each with inconsistent optimum concentrations and inconsistent units, so they are all expressed as high, medium and low concentrations in the text, with the specific action concentrations for each sample shown in the Table 1

Supplementary file2 (TIF 3685 KB) Effect of nine samples of different concentrations on the expression of (a) FLG and (b) CASP14 genes in HaCaT cells

Supplementary file3 (TIF 1505 KB) Effect of nine samples of different concentrations on the expression of KLK7 genes in HaCaT cells

Supplementary file4 (TIF 1571 KB) Effect of nine samples of different concentrations on the IL-6 secretion of HaCaT cells

Supplementary file5 (TIF 3847 KB) Effect of nine samples of different concentrations on the (a) IL-8 and (b) VEGF secretion of HaCaT cells
