# Supplementary figures and images for: Study of the protective effects of cosmetic ingredients on the skin barrier, based on the expression of barrier-related genes and cytokines
Source: Mol Biol Rep. 2021 Nov 19;49(2):989–95. doi: 10.1007/s11033-021-06918-5 (PMC8825566; doi:10.1007/s11033-021-06918-5)

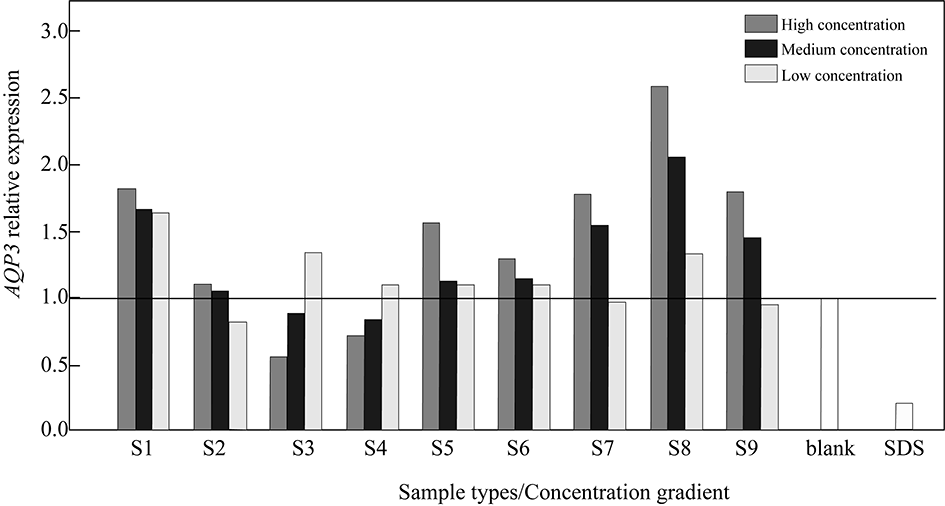

Supplement: Supplementary file 1 — Supplementary file1 (TIF 1662 KB) Effect of nine samples of different concentrations on AQP3 gene expression in HaCaT cells. The nine samples included both liquid and solid samples, each with inconsistent optimum concentrations and inconsistent units, so they are all expressed as high, medium and low concentrations in the text, with the specific action concentrations for each sample shown in the Table 1 [file 11033_2021_6918_MOESM1_ESM.tif]

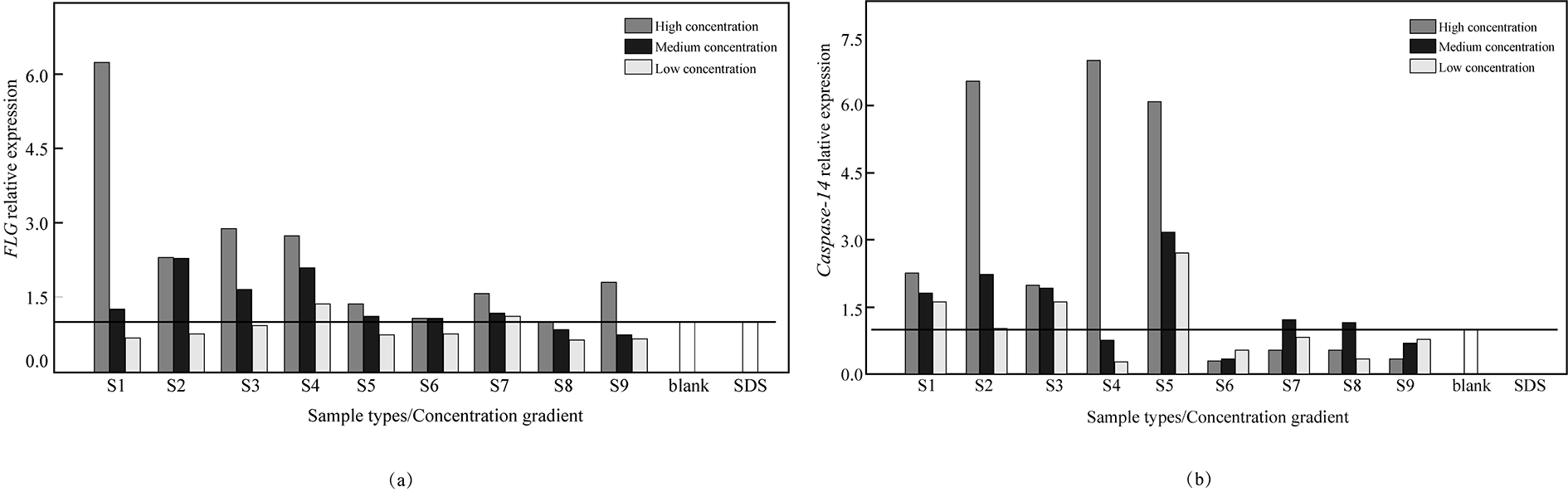

Supplement: Supplementary file 2 — Supplementary file2 (TIF 3685 KB) Effect of nine samples of different concentrations on the expression of (a) FLG and (b) CASP14 genes in HaCaT cells [file 11033_2021_6918_MOESM2_ESM.tif]

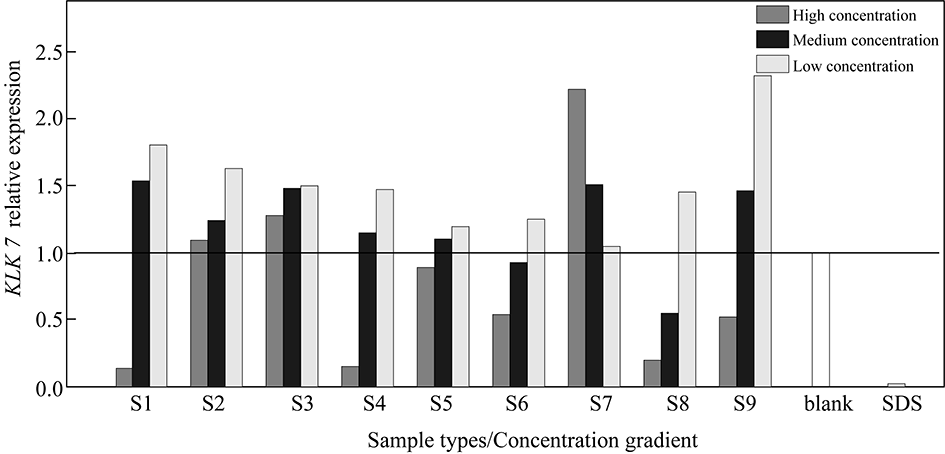

Supplement: Supplementary file 3 — Supplementary file3 (TIF 1505 KB) Effect of nine samples of different concentrations on the expression of KLK7 genes in HaCaT cells [file 11033_2021_6918_MOESM3_ESM.tif]

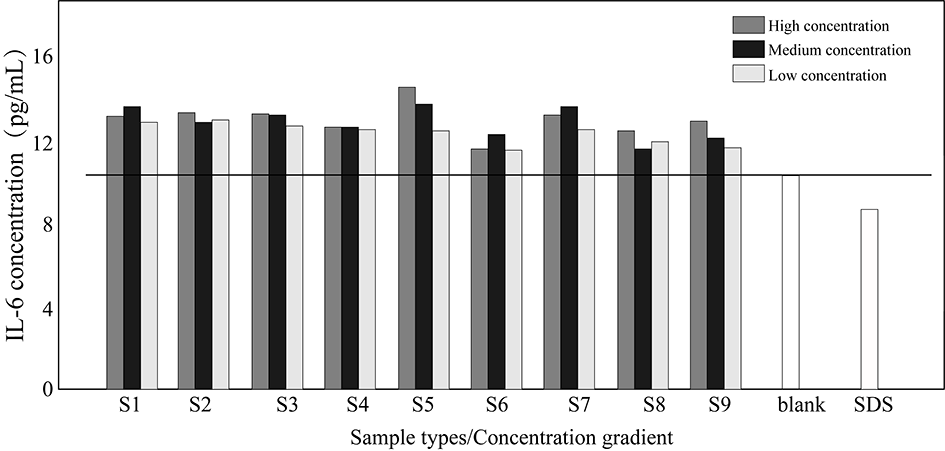

Supplement: Supplementary file 4 — Supplementary file4 (TIF 1571 KB) Effect of nine samples of different concentrations on the IL-6 secretion of HaCaT cells [file 11033_2021_6918_MOESM4_ESM.tif]

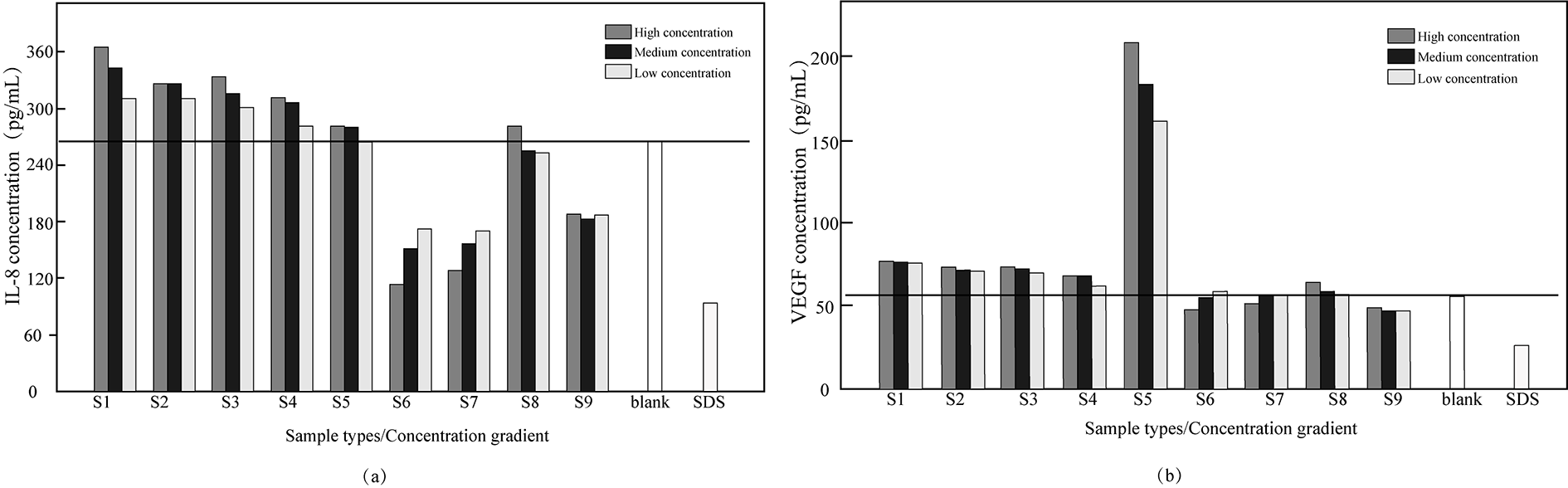

Supplement: Supplementary file 5 — Supplementary file5 (TIF 3847 KB) Effect of nine samples of different concentrations on the (a) IL-8 and (b) VEGF secretion of HaCaT cells [file 11033_2021_6918_MOESM5_ESM.tif]
